# An integrated pathology and ultrasonography-based simulation for training in performing kidney biopsy 

**DOI:** 10.5414/CN109267

**Published:** 2017-12-18

**Authors:** Shree G. Sharma, John M. Arthur, Stephen M. Bonsib, Kevin D. Phelan, Manisha Singh, Nithin Karakala, Kelly W. Bulloch, Vandana Dua Niyyar, Juan Carlos Q. Velez

**Affiliations:** 1 Arkana Laboratories, Little Rock, AR,; 2 Department of Pathology, University of Arkansas for Medical Sciences, Little Rock, AR,; 3 Division of Nephrology, Department of Medicine, University of Arkansas and Medical Sciences, Little Rock, AR,; 4 Division of Nephrology, Department of Medicine, Emory University School of Medicine, Atlanta, GA, and; 5 Department of Nephrology, Ochsner Clinic Foundation, New Orleans, LA, USA

**Keywords:** bedside ultrasound, graduate medical education, renal biopsy, procedure skill, simulation center, academic setting

## Abstract

Background: Medical practice trends and limitations in trainees’ duty hours have diminished the interest and exposure of nephrology fellows to percutaneous kidney biopsy (PKB). We hypothesized that an integrated nephrology-pathology-led simulation may be an effective educational tool. Materials and methods: A 4-hour PKB simulation workshop (KBSW), led by two ultrasonography (US)-trained nephrologists and two nephropathologists, consisted of 6 stations: 1) diagnostic kidney US with live patients, 2) kidney pathology with plasticine models of embedded torso cross-sections, 3) US-based PKB with mannequin (Blue Phantom™), 4) kidney pathology with dissected cadavers, 5) US-based PKB in lightly-embalmed cadavers, and 6) tissue retrieval adequacy examination by microscope. A 10-question survey assessing knowledge acquisition and procedural confidence gain was administered pre- and post-KBSW. Results: 21 participants attended the KBSW and completed the surveys. The overall percentage of correct answers to knowledge questions increased from 55 to 83% (p = 0.016). The number of “extremely confident” answers increased from 0 – 5% to 19 – 28% in all 4 questions (p = 0.02 – 0.04), and the number of “not at all confident” answers significantly decreased from 14 – 62% to 0 – 5% in 3 out of 4 questions (p = 0.0001 – 0.03). Impact of the imparted training on subsequent practice pattern was not assessed. Conclusion: A novel KBSW is an effective educational tool to acquire proficiency in PKB performance and could help regain interest among trainees in performing PKBs.

## Introduction 

Kidney biopsy is an invasive procedure designed to obtain essential diagnostic and prognostic information regarding the pathology and/or pathogenesis behind a disease process, with the overarching goal of impacting treatment. A kidney biopsy procedure involves obtaining a small piece (core) from the kidney with the use of a needle biopsy gun. The obtained tissue is submitted to a pathology laboratory for processing. It is then read by a pathologist who interprets the sample and generates a diagnostic report. 

The most commonly used method to obtain kidney tissue for diagnosis is the percutaneous kidney biopsy (PKB). Alternative approaches are also available, e.g., transjugular or transfemoral, transurethral, laparoscopic, or open kidney biopsy [1]. With the introduction of spring-loaded biopsy guns and ultrasound (US)-guided imaging technology, PKB can be easily performed at the bedside, with a relatively low rate of complications. For instance, major bleeding rates after a biopsy vary from 0.1 [2] to 12% [3, 4] depending on the baseline risk factors of the population. Life-threatening complications are seen in less than 0.1% of the cases [5]. Although some centers prefer US-based guidance for PKBs, an increasing number of centers around the United States now utilize computer tomography-based guidance as a routine modality. 

Upon retrieval of kidney biopsy specimens, the tissue is systematically processed and prepared for evaluation under light microscopy, immunofluorescence, and electron microscopy. To provide an accurate diagnosis, the pathologist must be provided with an adequate amount and quality of tissue. Each kidney has around a million nephrons [6], and the biopsy only samples a few representative glomeruli or nephrons. If 10% of the glomeruli in the kidney are affected by a disease, a sample containing 10 glomeruli will have 35% probability of missing the disease, while a sample with 20 glomeruli will have 12% probability of missing the disease [7]. Therefore, when focal disease is suspected, it is preferable to have a sample with 20 – 25 glomeruli [8] to render a correct diagnosis. A biopsy can be inadequate either due to a small sample size or due to predominantly noncortical tissue (e.g., medulla or fibrofatty tissue). For kidney biopsies, a 16G needle is considered optimal [9]. Although the adequacy of a biopsy sample is sufficient with both 14G and 16G needles, the complication rates in some series utilizing 14G needles has been reported to be higher than those with 16G needles [10], while in other series they are reported to be comparable [11]. On the other hand, the diagnostic yield and quality of biopsy cores might be better when utilizing 14G and 16G needles as compared to smaller 18G needles [12]. 

PKBs were initially performed exclusively by nephrologists. However, nowadays, a majority of these procedures are performed by interventional radiologists. This phenomenon is due to time constraints in current clinical practice combined with unattractive insurance reimbursement fee schedules [12]. In addition, inadequate training in the performance of PKB at many academic institutions appears to contribute to the ongoing trend towards abandonment of this procedure in nephrology. The suboptimal training in PKB during fellowship may be partly driven by diminished interest from the faculty themselves who are not comfortable performing the procedure, not to mention the current limitation of trainee duty hours. This, in turn, makes nephrology a less appealing specialty to internal medicine residents who are attracted to procedural specialties [13]. The Accreditation Council for Graduate Medical Education (ACGME) requires training of fellows in performance of native and transplant kidney biopsies, but no number is specified, and no criteria exist to assess performance [14]. Interestingly, performing a medical kidney biopsy is not part of the curriculum for interventional radiology [15]. In the interventional radiology literature, 18G needle is suggested for the core needle biopsy [16]. 

Thus, to address the existing gap and challenges in training in performance of PKBs, we conducted a dedicated workshop during the proceedings of a nephrology conference (KidneyCon 2016) at the main campus of the University of Arkansas for Medical Sciences Medical Center, in Little Rock, Arkansas. We hypothesized that an educational workshop that integrates training in kidney US and US-guided PKB, renal anatomy, and fundamentals of pathology and biopsy-specimen adequacy could improve the knowledge, level of confidence, and interest among trainees in performing this procedure. 

## Materials and methods 

### Workshop design 

**Figure 1.**
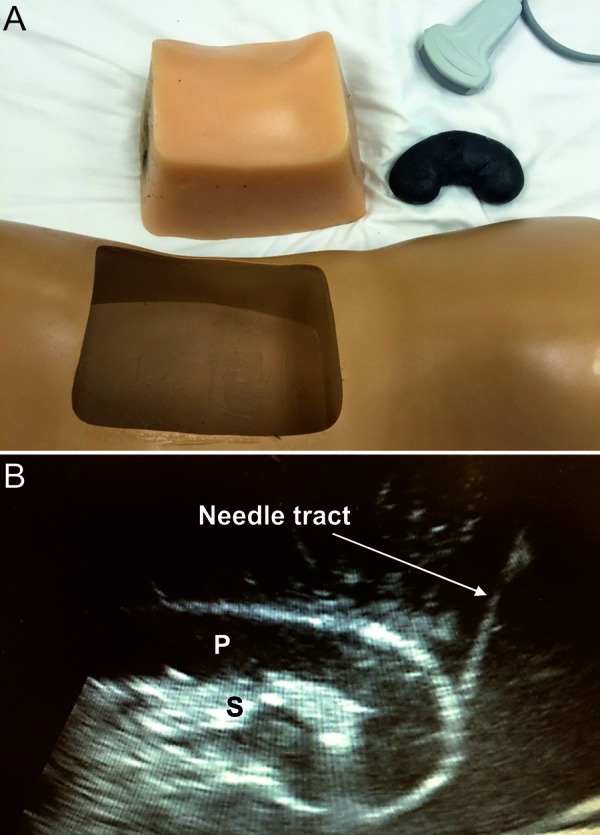
A: Display of the components of a commercially-available mannequin utilized in the fourth station of the Kidney Biopsy Simulation Workshop: insert of a model torso and a model kidney are shown. B: Image (sagittal view) obtained by ultrasound from a mannequin kidney (Blue Phantom™) during a real-time US-guided biopsy simulation, resembling the appearance of a native live kidney. Needle tract is depicted as it approaches the lower pole. S = renal sinus; P = renal parenchyma.

**Figure 2.**
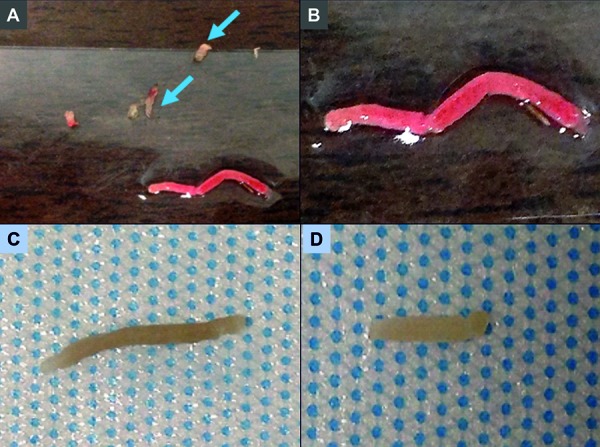
Images demonstrating appearance of kidney biopsy cores as shown to the Kidney Biopsy Simulation Workshop participants. A: Core biopsy of the renal cortex showing reddish-tan tissue. Fatty tissue in the periphery of the slide appears yellow (blue arrows). B: Higher magnification image of the kidney cortex. C: Kidney cortex after formalin fixation. D: Kidney medulla after formalin fixation.

We assembled a kidney-biopsy simulation workshop (KBSW) dedicated to training nephrology fellows and practicing nephrologists on the performance of PKB. Medical students and internal medicine residents were also encouraged to attend the conference to stimulate their interest in nephrology. The workshop was conducted within the University of Arkansas for Medical Sciences Medical Center facilities, lasted 4 hours, and was delivered by two US-trained nephrologists, two nephropathologists, and one anatomist. It was conducted on the day preceding the KidneyCon 2016 conference. Participants were divided into 3 groups and rotated through 6 modules/stations:

*1) Diagnostic kidney US with simulated patients:* Under the guidance and supervision of two nephrologists proficient in renal US, hands-on US of the kidneys and bladder were performed on simulated patients (healthy individuals). The fundamentals of ultrasonography as well as techniques for performing diagnostic and procedural US were briefly reviewed. All US-based examinations were conducted utilizing a General Electric (GE™) LOGIQ P9 (USA) device (GE Healthcare, Chicago, IL, USA). Each participant was given an opportunity to familiarize themselves with the machine and the probe and received hands-on training in performing the procedure. We paid particular attention to visualizing the kidneys in ventral decubitus resembling the most commonly used position for PKBs. Participants were taught how to distinguish the renal parenchyma from the renal sinus, how to identify the lower pole of the kidney, and how to recognize the variation in kidney position associated with respiration.
*2) Kidney anatomy with torso cross-sections:* Plasticine models of embedded cross-sections of the human body at abdominal level were displayed. Participants reviewed the models starting at the cross-section just above the adrenal gland and continuing to the lower pole of the kidney. The participants could appreciate the intra-abdominal and retroperitoneal organs and their relationship to the kidneys from one pole to the other. A handmade diagram was also given to the participants. They were quizzed informally prior to being given a labeled copy of a drawing of the anatomical relations of the kidneys in order to prime for the best learning experience.
*3) Kidney anatomy with dissected cadavers:* Three dissected cadavers were displayed. The first cadaver was dissected prone (lying face down). The abdominal wall was cut in layers, and the kidney was exposed for the viewing of the posterior relationships. The second cadaver kidney was exposed supine (face up) for the anterior relations. In the third cadaver, all the abdominal organs were removed, and the kidney was exposed in the retroperitoneal space, and its relationship to the vertebral column was shown.
*4) US-based PKB simulation with mannequin (Blue Phantom™)*: At this station, we utilized a mannequin to perform a hands-on PKB simulation (Figure 1A). The model kidney can be easily localized within the mannequin in a prone position with a curvilinear probe, and the generated images closely resemble those of real kidneys (Figure 1B). A handout was provided to the participants explaining the procedure along with indications, contraindications, and risks for complications, and they were instructed on the steps of the PKB procedure. The participants were taught how to hold and maneuver the kidney biopsy gun and the US probe, and had ample time to practice on the mannequin. The model kidney does not allow retrieval of kidney cores, therefore, we did not assess adequacy of sampling in this station. The techniques of real-time guidance as well as site-marking were demonstrated. Handouts detailing all the steps needed to safely perform a PKB were distributed to the participants.
*5) US-based PKB simulation in lightly-embalmed cadavers:* PKBs were performed on lightly-embalmed cadavers positioned in ventral decubitus. Light embalming technique was done per a published protocol [17]. Two cadavers of different body habitus and body mass index were used. The attendees were given hands-on experience, and each participant was allowed to perform the biopsy. Two different standard techniques of kidney biopsy were taught to the participants: *5A) US-based site marking only:* In this technique, the lower pole of the kidney was visualized, and the corresponding site for needle incision was marked after verification in two planes. The probe was kept at an angle perpendicular to the bed so that it was easily reproducible and so that the needle insertion could be directly inserted along the same plane without real-time guidance. The depth of the cortex of the kidney was also noted on the US. After marking the site, a small incision was made with a scalpel. Then, the biopsy needle was introduced at the same angle, up to the previously-marked depth. The biopsy gun was then fired, and the sample retrieved; *5B) Real-time US-guided:* In this technique, the lower pole of the kidney was also visualized. In this case, the angle for the needle insertion was not perpendicular to the skin, but almost perpendicular to the probe to maximize visualization of the needle as it is inserted through the skin, across the subcutaneous and muscle tissue, and towards the renal capsule. At that point, the biopsy gun was fired and the sample retrieved. Participants were allowed to make several attempts to obtain a biopsy core and were instructed to take one of their obtained cores to the following station of microscopic examination of adequacy.
*6) Tissue retrieval adequacy examination by microscope and smartphone camera:* Participants received instruction on how to assess the adequacy of their samples. A tissue core was spread out on a slide and examined both macroscopically and microscopically. A standard light microscope was available and set up for examination of the tissue cores under bright field. The participants were shown how to distinguish cortex (tan in appearance) from medulla (whitish/grayish, not as tan as compared to the cortex) and the fat/renal capsule (yellowish and very stringy) and were taught how to identify glomeruli within the tissue core. In addition, photographs of the kidney specimens were obtained using a smartphone camera (Figure 2), and the above-mentioned nuances were further explained to the attendees. The advantage of zooming the photograph with a smartphone was demonstrated, a tool that can be utilized when a microscope is not readily available in the procedural suite.

### Ethical considerations 

The administration of the 4-hour course, the utilization of cadavers for the third and fifth station, and publication of the proceedings and results of the survey were approved by the institutional Ethics Committee. The identity of the cadavers and healthy volunteers remains strictly confidential. 

### Survey

Immediately before and after the KBSW, i.e., within 15 minutes of the beginning and end of the workshop, a survey was conducted to assess its impact on improving knowledge and procedural confidence. The survey comprised 10 questions: 6 multiple-choice questions assessing knowledge acquisition and 4 five-scale questions assessing procedural confidence gain. An additional question was included to inquire about the likelihood that the participants would be recommending the workshop to other colleagues. 

### Statistics 

Comparison of proportions were performed by χ^2^-test. Comparison of means of continuous variables were performed by t-test. A p-value < 0.05 was considered significant. 

## Results 

**Figure 3.**
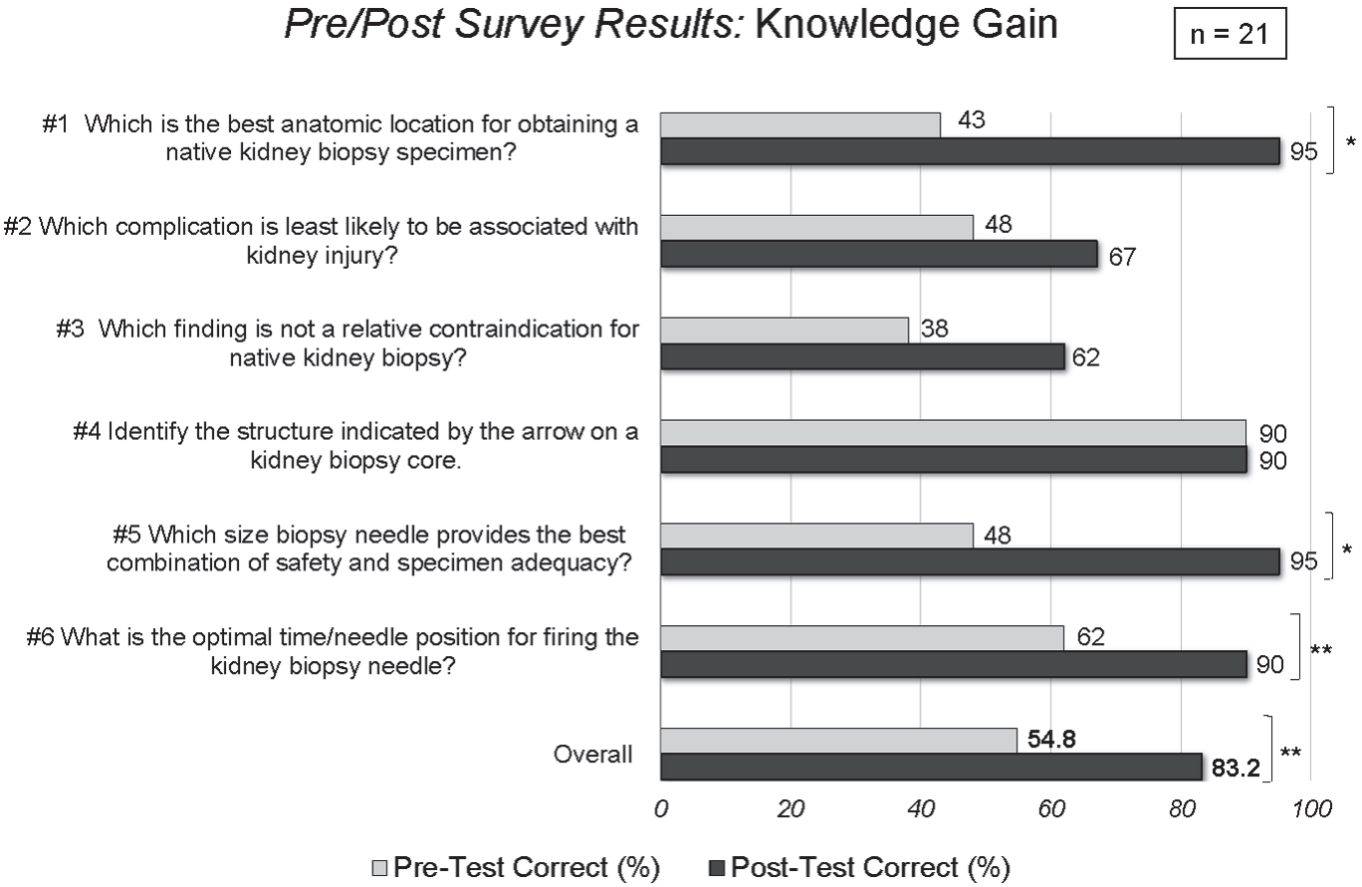
Results of survey demonstrating increase in knowledge pertinent to kidney biopsy performance acquired after the workshop; *p < 0.0001, **p < 0.05, for comparison of means.

21 physicians (4 nephrologists, 17 trainees) participated in the KBSW and completed the pre- and post-surveys. No participant refused to complete the survey. On knowledge-based questions, the percentage of correct answers increased numerically for 5 of the 6 questions (Figure 3). Three (50%) individual questions reached statistically-significant improvement (from 43%, 48%, and from 62 to 95%, 95%, and 90%, respectively, p = 0.0003 – 0.04); 1 question already had 90% correct answers in the pretest. The overall percentage of correct answers increased from 55 to 83% (p = 0.016). 

**Figure 4.**
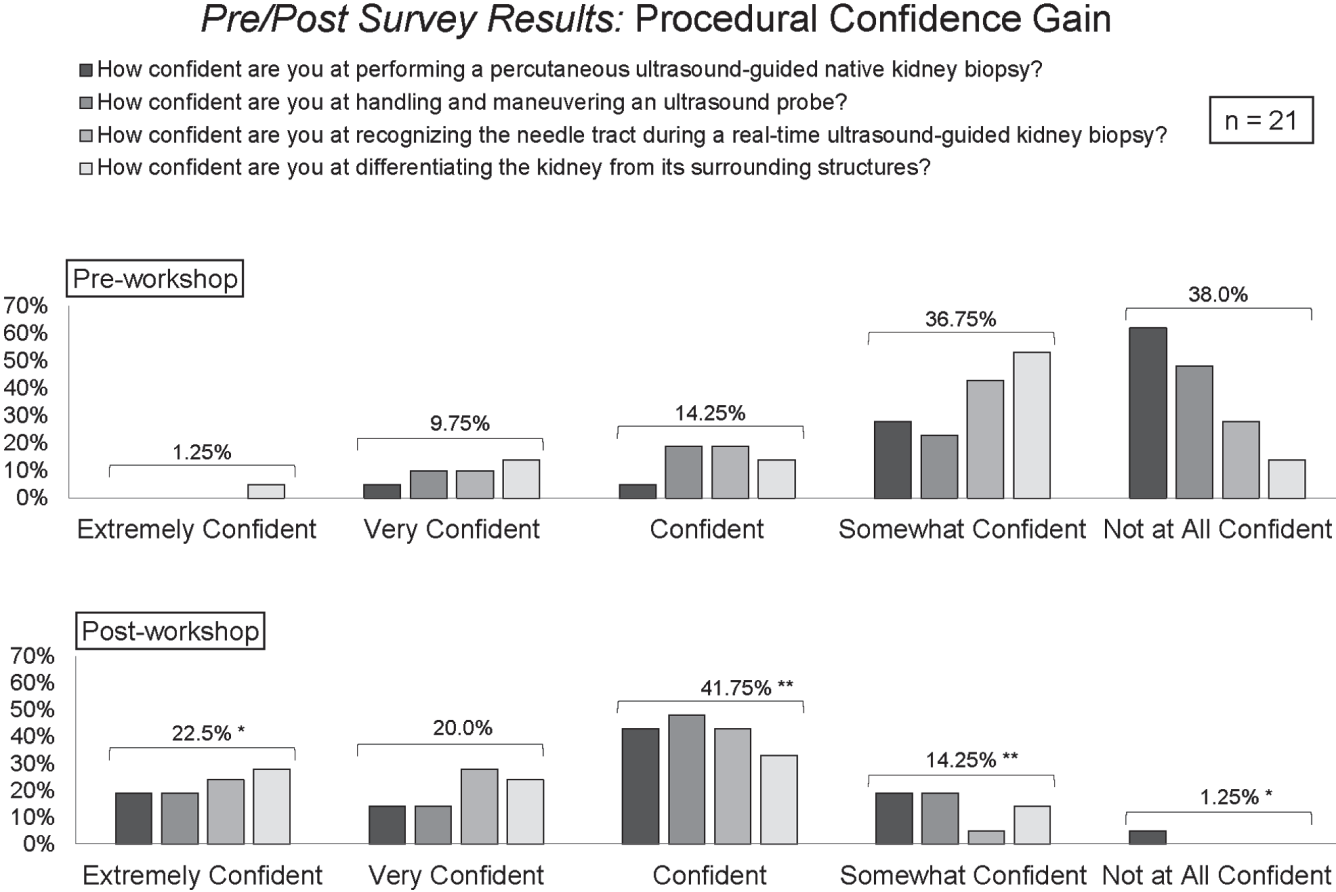
Results of survey demonstrating increase in procedural confidence gained after the workshop; *p < 0.001, **p < 0.05, for comparison of means.

In terms of the questions addressing procedural confidence, the number of “extremely confident” answers increased from 0 – 5% (mean 1.25%) to 19 – 28% (mean 22.5%) in all 4 questions (p = 0.02 – 0.04), and the number of “not at all confident” answers significantly decreased from 14 – 62% (mean 38%) to 0 – 5% (mean 1.25%) in 3 out of 4 questions (p = 0.0001 – 0.03) (Figure 4). The percentage of answers “somewhat confident” and “confident” also changed significantly, whereas the number of “very confident” answers did not significantly change. 

Furthermore, 67% of participants stated that they were “extremely likely” to recommend the KBSW to others. 

## Discussion 

The goal of our simulation workshop was to increase knowledge of, and confidence in, all aspects of performing a PKB with the hope of promoting its resurgence in nephrology training programs as well as among practicing nephrologists. The strategy to achieve this goal employed a blended learning approach. Pre- and post-workshop surveys demonstrated a significant increase in the knowledge-based questions and significantly improved the confidence level in performance of the procedure. These improvements may increase the likelihood that participants will choose to perform PKBs in their clinical practice. Moreover, it may potentially improve rates of tissue adequacy and, therefore, the clinical utility of the biopsy. However, such benefit would require long-term evaluation of the workshop. 

Over the past decade, PKBs have increasingly moved from the domain of nephrology to interventional radiology, even though there is lack of dedicated training for the performance of PKB within most radiology training programs. In parallel with these changes, nephrology trainees are receiving less training and acquiring less experience with the PKB procedure. The variability in technical resources and dwindling biopsy procedure training may deprive fellows of the necessary experience to confidently and safely perform a PKB [19]. In a well-structured fellowship training program, PKB is safe and an important aspect of training [18]. Interestingly, fewer complication rates have been reported for trained nephrologists as compared to radiologists [19]. However, other studies suggest comparable complication rates [20]. The likelihood of complications appears to correlate with the frequency of procedures undertaken. In centers performing less than 30 biopsies per year, the complication rates are higher compared to centers performing more procedures [21]. Thus, improving or supplementing training in PKB performance may help reduce the risk for complications when PKBs are performed by inexperienced practitioners and/or in low-volume facilities. 

An optimal outcome for the procedure is to maximize adequacy of biopsy samples while minimizing complication rates [12]. The issues can be addressed with proper training. Some simulation trainings have shown success at delivering effective instruction [4, 22]. In the course delivered by Oliver et al. [4], the candidates were taught PKB on frozen cadaveric tissue, which resulted in positive feedback from the attendees. Similarly, in a course delivered by Dawoud et al. [23], nephrology fellows were trained using a developed simulation tool, which consisted of a porcine kidney inserted under a turkey breast. After simulation training, the fellows showed increased confidence in performing kidney biopsies. Moreover, the authors reported a reduction in severity of biopsy-associated bleeding complications. Unlike these reports, our workshop provided a comprehensive overview of anatomy, diagnostic radiology, biopsy technique, and adequacy of the biopsied tissue in order to provide participants a well-rounded overview of the important elements of the procedure, including the retrieval of adequate tissue specimens. In addition, the novelty of our approach was that it consisted of two different simulation layouts, a mannequin-based and a cadaver-based model. 

Many hospitals do not have a renal pathologist physically present at their facility. As a result, hospitals are forced to utilize the services of a distant reference pathology laboratory. A significant caveat of that mechanism is the inability to unequivocally confirm the adequacy of the specimen prior to shipment. If the obtained biopsy specimen is not adequate for diagnosis, the treating nephrologist may not be able to make a treatment decision in a timely manner. Furthermore, if the procedure needs to be repeated, the patient has to be subjected to additional risk for the complications inherent to a PKB. Determination of specimen adequacy at the time of the procedure is therefore critical and dictates the number of passes performed and the ability to establish a pathological diagnosis. The KBSW participants received instruction on assessment of tissue adequacy at the bedside, specifically how to distinguish cortex from medulla and fibrofatty tissue. This was feasible during the workshop since tissue obtained from the lightly-embalmed cadavers is similar to freshly-obtained biopsy tissue. In live kidneys, red blood cells are visible in the glomeruli and vessels, permitting participants to distinguish cortex from medulla more easily. Red blood cells were not visible in the embalmed cadavers or in the fixed biopsy tissue. 

A limitation of the KBSW is the inability to simulate the positional variation of the kidney during respiration by either the mannequin-based or the cadaver-based models. However, we addressed this aspect of the procedure in the live US station. In addition, because we did not collect data related to performance of PKB during the rest of the fellowship training and/or clinical practice of the participants, we are unable to assess the impact of our workshop and training approach on subsequent behavior or change in actual medical practice. Further, we did not collect feedback specific to any of the 6 stations. Therefore, we cannot determine whether some stations were more influential or effective than others. In regards to using metrics for success of the workshop, we did not record the number or proportion of adequate kidney specimens obtained by the participants during the cadaver-based station. However, all participants were able to verify a successful retrieval of at least one adequate specimen. 

In conclusion, a KBSW (which we have now renamed Kidney Biopsy Academy) utilizing US-based training on mannequins and cadavers as well as diverse anatomic displays of the regional anatomy of the kidney is an effective educational tool to increase proficiency in PKB performance. This innovative approach could bring the PKB back into the domain of nephrology while attracting more trainees to perform PKBs after graduation. 

## Funding 

None. 

## Conflict of interest 

No conflict of interest related to the content of this manuscript. However, J.C.Q.V is a member of Speaker Bureau and Advisory Board for Mallinckrodt Pharmaceuticals and Speaker Bureau for Otsuka Pharmaceuticals (drugs related to those affiliations are not discussed in the manuscript).
